# Baijiu–Peanut Pairing In Vitro and In Vivo: The Decreased but Prolonged Aftertaste of Baijiu Under the Effect of Mouth Coating Formed by Peanut Lipid

**DOI:** 10.3390/foods14030423

**Published:** 2025-01-28

**Authors:** Lu Chen, Jinyuan Sun, Yanyan Zhang, Hehe Li, Dongrui Zhao, Bowen Wang, Xingqian Ye, Baoguo Sun

**Affiliations:** 1 College of Biosystems Engineering and Food Science, National–Local Joint Engineering Laboratory of Intelligent Food Technology and Equipment, Zhejiang Key Laboratory for Agro-Food Processing, Zhejiang University, Hangzhou 310058, China; chenlu00318@163.com; 2 Key Laboratory of Brewing Molecular Engineering of China Light Industry, Beijing Technology and Business University, Beijing 100048, China; xyzhehe@126.com (H.L.); zdrui6789@sina.com (D.Z.); wangbw@btbu.edu.cn (B.W.); sunbg@btbu.edu.cn (B.S.); 3 Department of Flavor Chemistry, Institute of Food Science and Biotechnology, University of Hohenheim, 70599 Stuttgart, Germany; yanyan.zhang@uni-hohenheim.de

**Keywords:** baijiu, aroma compounds, aftertaste, food pairing

## Abstract

Baijiu–peanut pairing is a popular food combination, wherein peanuts, particularly their lipid component, possess the potential to influence the flavor of baijiu and consequently modulate its aftertaste. To reveal the role of peanut lipid within this system, headspace experiments and equilibrium constant measurements were conducted. It was determined that peanut lipids are capable of inhibiting the release of flavor substances in baijiu, and this inhibitory effect is concentration-dependent. Molecular dynamics simulation results show a strong interaction between peanut lipids and ethanol (E binding = −2.181 kJ/mol), which weakens the binding energy between ethanol and compounds, resulting in 1 + 1 < 2 effect. The Intraoral SPME experiment revealed the retention effect lipid-coating on the aroma release of baijiu. The flavor substances retained in the oral coating can continuously contribute to the aftertaste perception of baijiu. Peanut lipids, leveraging the Marangoni effect of ethanol, play a role in prolonging the aftertaste of baijiu, either through direct or indirect ways. This study provides a scientific basis for understanding the complex flavor experience in the food pairing process.

## 1. Introduction

Food flavor is one of the key attributes of food and plays a decisive role in consumer preference. Currently, the flavor research of distilled spirits mainly focuses on its objective aroma composition, while little attention is paid to its flavor performance during the eating and drinking process [1]. For example, for distilled spirits, such as brandy and vodka, adding ice and mixing cocktails will affect consumers’ flavor perception. However, when it comes to baijiu, the national liquor of China, consumers mostly choose to drink it neat and pair it with food. Peanut is the most common accompanying food. Baijiu is made from various grains through microbial fermentation, distillation, blending, and aging [1]. Its aroma substance composition is extremely rich, and more than 3000 flavor substances have been discovered so far [2]. Rich aroma and long aftertaste are the typical flavor characteristics of baijiu. The aftertaste involves a complex oral processing process, which has not been fully studied so far. In the previous stage, we found that, in the baijiu–peanut pairing, peanuts can selectively affect the release pattern of baijiu flavor substances in the oral cavity, bringing the possibility of regulating the aftertaste of baijiu [2,3]. Nearly half of the peanut components are lipids [4]. Together with water and ethanol, they are the food components that most significantly affect the release intensity of flavor compounds [5]. In the baijiu–peanut system, these three major influencing factors coexist, and their combined effect on the release of baijiu flavor is worthy of in-depth exploration [6].

Most of the flavor substances in baijiu are polar compounds with lipophilicity. Lipids can significantly change their release intensity [7]. Under the influence of lipids, the release intensity of octanoic acid and γ-decalactone is reduced by 60 times, and the threshold of 2,4-decadienal is increased by 600 times [8]. In the oral environment, lipids can also significantly affect the flavor performance of compounds. Van Ruth found that the reduction in lipid content in the oral environment can promote the release intensity of compounds such as alcohols, ketones, esters, aldehydes, terpenes, and sulfur-containing compounds, and the degree of influence is related to the type and chain length of the compounds [9]. Lipids and aroma compounds can interact through hydrophobic interaction forces, van der Waals forces, hydrogen bonds, etc., thus affecting the volatilization process of substances [7].

In the dynamic process, the influence of lipids on food flavor is more obvious [6]. The lipid content of food will not only affect the perceived intensity but also the time distribution of flavor. Researchers have found that when eating lipid-containing food, lipids or lipid substances can form an oral coating on the tongue under the pressure of the tongue on the soft palate, affecting the aftertaste of the food [10]. The barrier formed by the oral coating limits the access of taste substances to taste receptors. For volatile flavor substances, they must first be released from the lipid-containing system before they can enter the headspace to form retronasal aroma. Therefore, lipid-soluble compounds are perceived later than water-soluble compounds [11]. Studies on the surface of the pig tongue have found that the amount of residual food on the oral surface will increase with the increase in fat content [12]. The fat in cheese forms an oral coating that can delay the transfer of aroma compounds and slow down their release in the aftertaste, so that the flavor substances are slowly released in the post-swallowing stage, prolonging their flavor perception [13]. The oral coating formed by peanut lipids during the eating process may be one of the main ways that peanuts affect the aftertaste of baijiu.

Ethanol is an important matrix component in baijiu and is a special food matrix that distinguishes the flavor research of alcoholic beverages from other foods. In a static environment, ethanol can inhibit the release intensity of non-polar compounds and increase the threshold of compounds. In a dynamic system, the presence of ethanol helps maintain the headspace concentration of volatile compounds, known as the Marangoni effect, which makes the flavor substances in the ethanol solution continuously release at a constant intensity, mainly due to the surface activity of ethanol [14,15]. In the oral system, the ethanol concentration can significantly affect the interaction between the aroma compounds of baijiu and the components of saliva [16]. For example, ethanol can denature salivary mucin, which in turn promotes the release intensity of esters. Under the joint mediation of ethanol and lipids, how is the flavor of baijiu affected? And in the complex oral environment, how does the oral coating formed by peanut lipids affect the aftertaste of baijiu? In this study, the influence of peanut lipids on the important flavor substances of baijiu in an ethanol environment was first studied through headspace experiments and equilibrium constant measurement experiments, and the energy change in the interaction between substances was studied using molecular dynamics simulation. Then, on this basis, the Intraoral SPME method was used to investigate the influence of the oral coating formed by peanut lipids on the aftertaste of baijiu in the real oral cavity.

## 2. Materials and Methods

### 2.1. Experimental Materials

The peanut lipid used in the experiment was purchased from Yuanye Bio-Technology Co., Ltd. (Shanghai, China), with a purity of 97%. The peanut lipid for the Intraoral SPME experiment was food-grade peanut lipid produced by Hujihua Food Co., Ltd. (Qingdao, China). The strong-flavor baijiu was produced by the Gujing Group (Bozhou, China), with an alcohol content of 53% ABV. Before the experiment, the compounds in baijiu were screened to ensure that they could be dissolved in the experimental solution and stable peak areas could be obtained. In total, 19 target aroma compounds were screened according to the compound types and chain lengths, as shown in Table 1. The compound standards were purchased from J&K Scientific Co., Ltd. (Beijing, China) and Macklin Co., Ltd. (Shanghai, China), with a purity of 98–99.5%. Ethanol was purchased from J&K Scientific Co., Ltd. (Beijing, China), with a purity of 99.7%. Milli-Q distilled water was used for the experiment.

### 2.2. Effect of Peanut Lipid on the Release Intensity of Baijiu Flavor Compounds

HS-SPME was used to detect the effect of peanut lipid on the release intensity of baijiu aroma compounds in aqueous solution and 53% ABV solution. The SPME fiber with DVB/CAR/PDMS adsorption material was used for the experiment. In 20 mL headspace vials, solutions with water and 53% ABV ethanol aqueous solution as the matrix were prepared, respectively, and 200 μL of the high-concentration mixed stock solution containing the target compounds (the solvent was ethanol) was added. The final concentrations of the compounds are shown in Table 1. Peanut lipid, at 4%, was added to the experimental group. All samples were vortexed for 8 min for full emulsification and mixing, equilibrated at 50 °C for 5 min, and vacuum-absorbed for 10 min. Each group of experiments was repeated 3 times.

### 2.3. Equilibrium Constant Detection Experiment

To study the concentration dependence of the effect of peanut lipid on the release intensity of baijiu flavor substances, the equilibrium constants of the compounds were measured when the addition amount of peanut lipid in 53% ABV solution was 0%, 4%, and 8%, respectively. The SPME method was the same as that in 2.2. The measurement method of the compound equilibrium constant was appropriately modified with reference to the method in the literature [17,18]. The partition coefficient (K) was determined by studying different phase ratios (*β*) in the headspace vial. The phase ratio is the ratio of the volume of the headspace to the volume of the sample solution:

(1) $$\beta =\frac{V_{G}}{V_{S}},$$

where $V_{G}$ is the gas phase volume and $V_{S}$ is the liquid phase volume.

A linear equation was drawn between the reciprocal of the peak area (1/*A*) and the phase ratio (*β*):

(2) 1A=aβ+b.

The partition coefficient is defined as the ratio of the slope (*a*) to the intercept (*b*) of this equation.

Solutions with different peanut fat contents were transferred to 20 mL headspace vials in gradient volumes (0.5 mL, 1 mL, 2 mL, 4 mL), and the phase ratios of each vial were 39, 19, 9, and 4, respectively. Other conditions were the same as those in the HS-SPME experiment. To ensure the repeatability and linearity of the experimental data, the experiment was repeated 5 times.

### 2.4. Intraoral SPME Experiment

A total of 6 test subjects were recruited, aged 24–30, 3 males and 3 females, with healthy oral status and who were non-smokers. They had all drunk baijiu and had some experience in its sensory evaluation. The oral cavity was cleaned, and the subjects fasted for 1 h before the experiment. Each test subject had been trained before the experiment to fully understand the sampling procedure. The study was conducted in accordance with the Declaration of Helsinki, and the protocol was approved by the Ethics Committee of Beijing Technology and Business university on 24 May 2023. All subjects participated in the experiments both with and without lipid.

Intraoral SPME was used to detect the aftertaste intensity of baijiu in vivo, with and without peanut lipid. The method and experimental conditions follow the relevant baijiu research experiment [19,20]. An aromatic baijiu added with food-grade compound standards was used for the experiment to obtain stable experimental results, and the additional amounts of the compounds are shown in Table 1. During the experiment, the test subjects needed to put 5 mL of the aromatic baijiu into their mouths, rinse for 15 s, and then spit it out. After spitting, the SPME fiber equipped with a handheld bracket was placed in the oral cavity at 0 s, 30 s, 60 s, 90 s, and 120 s. During the extraction process, the lips wrapped around the metal casing of the extraction device and remained closed, forming a closed and stable system for the extraction experiment. The extraction time was 2 min. After extraction, the extraction fiber was immediately transferred to the GC—MS injection port for thermal desorption. The extraction fiber was baked at 250 °C for 10 min between each experiment to avoid compound residues. In the experimental group with peanut lipid participation, the test subjects needed to rinse with 5 mL of peanut lipid for 15 s, spit it out, and then rinse with the flavored baijiu sample. Each test subject repeated the experiment 3 times.

### 2.5. GC-MS Operating Conditions

The gas chromatograph 7890B-5975 (Agilent Co., Ltd., Santa Clara, CA, USA) equipped with a DB-FFAP chromatographic column (60 m × 250 μm × 0.25 μm) was used. The injection port temperature was 250 °C, the carrier gas was helium, and the flow rate was 1.0 mL/min. The gradient temperature program of the gas chromatograph was as follows: the initial column temperature was 40 °C, maintained for 4 min, then increased to 220 °C at a rate of 8 °C/min and maintained for 5 min. After the temperature program ended, it was run at 240 °C for 5 min to ensure that all the compounds in the chromatographic column had been eluted. The interface temperature of the mass spectrometer was 250 °C, the ion source temperature was 230 °C, and the EI voltage was 70 eV.

### 2.6. Molecular Dynamics Simulation

Gromacs combined with Molecular Mechanics/Poisson–Boltzmann Surface Area (MM-PBSA) was used to simulate the interactions between baijiu flavor substances, peanut lipid, and ethanol molecules, and to study the changes in their interaction energies. The files of baijiu flavor substances and lipid molecules were obtained from http://atb.uq.edu.au/ (accessed on 26 November 2024). The force field was modified on the basis of the gromos54a7 force field. Packmol was used to construct a box of 6 nm × 6 nm × 6 nm, and a 53%ABV ethanol aqueous solution system was set up. According to the composition ratio of peanut lipid, 4 OOL molecules, 2 OLL molecules, and 2 POL molecules (the letters represent the types of triglyceride side chains, O—oleic acid, L—linoleic acid, P—palmitic acid) were placed in the box [4]. To compare the differences in interaction forces, 2 molecules of each selected baijiu flavor substance were placed in the system. The simulation time was 50,000 ps. Gmx-mmpbsa was used to process the interaction energies between molecules.

### 2.7. Data Processing

GraphPad Prism 8 was used for significance analysis, data collation, and drawing result graphs. XLSTAT 14.0 was used for PCA analysis. Gromacs was used for molecular dynamics simulation, and VMD was used to visualize the simulation results and perform polarity analysis of the molecular motion trajectories. APBS 1.5 was used to call the gmx—mmpbsa software to calculate the changes in the binding energy between molecules during the molecular dynamic simulation process.

## 3. Results and Discussion

### 3.1. Influence of Lipid on the Release of Baijiu Flavor Substances

**Figure 1:**
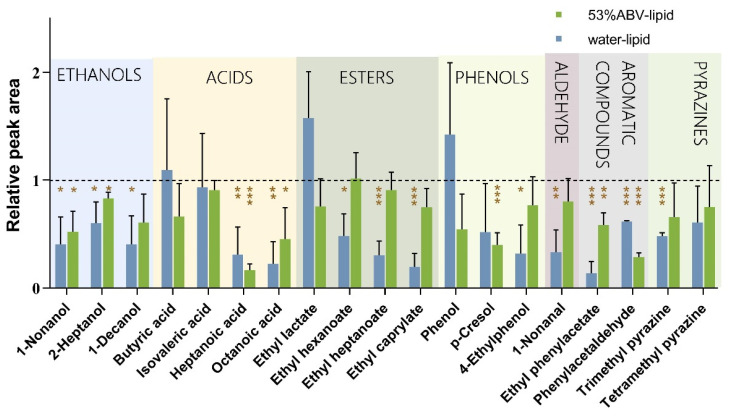
The influence of peanut lipid on the aroma release of baijiu components. The “*” shows a significant difference in the release intensity of compounds after adding peanut lipid compared to the blank control group. The “*” represents *p* < 0.05, “**” represents *p* < 0.01, and “***” represents *p* < 0.001 in the multiple *t*-test.

HS-SPME was employed to investigate the impact of peanut lipid on the release intensity of baijiu aroma substances in water solution and 53% ABV solution (Figure 1). Thanks to the small amount of ethanol in the compound stock solution as the solvent, along with a prolonged vortex homogenization and shortened SPME equilibrium extraction time, most compounds had an RSD < 15%. The experimental results demonstrated that in the water solution, peanut lipid promote the release of butyric acid, ethyl lactate, and phenol (*p* > 0.05; log *p* < 1.5 and water solubility constant > 1.0, thus being hydrophilic). For compounds with log *p* > 1.5, lipid reduced their release, and for those with log *p* > 1.7 and water solubility constant > 1.0 (except for p-cresol), the release was significantly inhibited, suggesting that the influence is associated with hydrophobicity.

In comparison with the water solution, the release intensities of compounds in the 53% ABV solution decreased. Notably, the releases of nonanol, 2-heptanol, heptanoic acid, octanoic acid, 4-methylphenol, ethyl phenylacetate, and phenylacetaldehyde were significantly inhibited by peanut lipid, indicating an interaction between peanut lipid and baijiu flavor substances. Except for phenylacetaldehyde, p-cresol, heptanoic acid, and isovaleric acid, the inhibitory effect of lipid on compounds in 53% ABV–lipid solution was weakened compared to the aqueous solution. The decreases in release intensities of 1-decanol, ethyl hexanoate, ethyl heptanoate, ethyl octanoate, 4-ethylphenol, nonanal, and trimethyl pyrazine under the influence of the lipid were no longer statistically significant. Both the lipid and ethanol can inhibit the release intensity of the compounds by increasing their hydrophobicity, but there is no additive effect after mixing, resulting in 1 + 1 < 2. Ethanol and lipid have an antagonistic effect on the release of aroma compounds.

**Figure 2:**
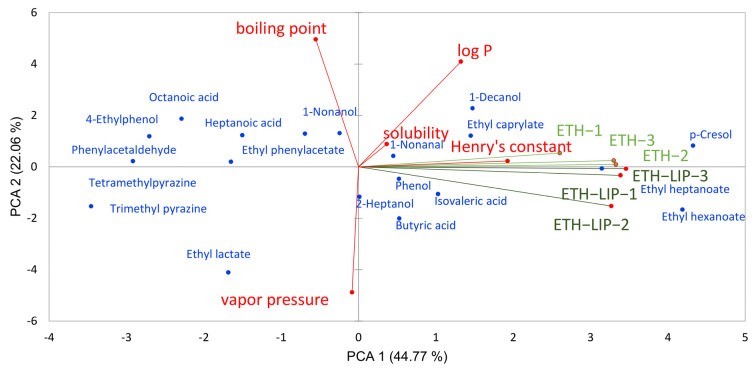
The PCA analysis of the influence of peanut lipid on baijiu flavor.

To explore the contribution of the physicochemical properties of the compounds to the influence of peanut lipid on baijiu flavor in 53% ABV solution, PCA was used to reduce the dimension of the experimental data (Figure 2). The data before and after adding the lipid were separated by PCA1, which correlated positively with the log *p* value and boiling point of the compounds and negatively with their vapor pressure. The boiling point of the compound relates to its intramolecular hydrogen bonds number, polarity, molecular weight, and volume of the compound. Log *p* can characterize hydrophobicity and solvent affinity, while vapor pressure indicates volatility. These properties determine the release pattern of baijiu aroma compounds under peanut lipid.

The equilibrium constant indicates the release intensity of the compound in the system, closely related to its solubility (Table 2). When the solubility of the compound is high, the volatility decreases and the equilibrium constant decreases. Compared with the water solution, the equilibrium constant of the compound in the 53% ABV solution decreases. After adding different concentrations of lipid to the 53% ABV solution, the equilibrium constants of most compounds showed a significant decreasing trend and were concentration-dependent. The change in the equilibrium constant of the compound in the 53% ABV solution and the water solution was not significant (Wilcoxon test, *p* > 0.05), while the 4% and 8% (*v*/*v*) lipid in the 53% ABV solution changed the constant significantly (*p* < 0.05). This indicates that the retention effect of peanut lipid is stronger than that of ethanol.

### 3.2. Molecular Dynamics Simulation of the Interaction Between Lipid Molecules and Ethanol Molecules

To clarify the influence mechanism of lipid molecules on the release of aroma compounds in 53%ABV solution, a molecular dynamics simulation system was constructed. Since too many molecules will bring excessive computational load and cause the system to crash, heptanoic acid, ethyl hexanoate, and ethyl lactate were selected as representative compounds. Ethyl lactate is hydrophilic (log *p* = −0.19) and shows unique release behaviors in the interaction with saliva [16], and the dynamic drinking process of baijiu under the effect of peanut [2,3,21]. Here, its release was promoted by peanut lipid in water but inhibited in 53% ABV solution. Ethyl hexanoate is highly concentrated in baijiu. Its release intensity was significantly inhibited by peanut lipid in water, but not in the 53% ABV solution. Heptanoic acid, a main acid in baijiu, had its release intensity notably reduced by peanut lipid in the water solution, and the inhibitory impact of peanut lipid on it was further augmented in 53% ABV solution.

**Figure 3:**
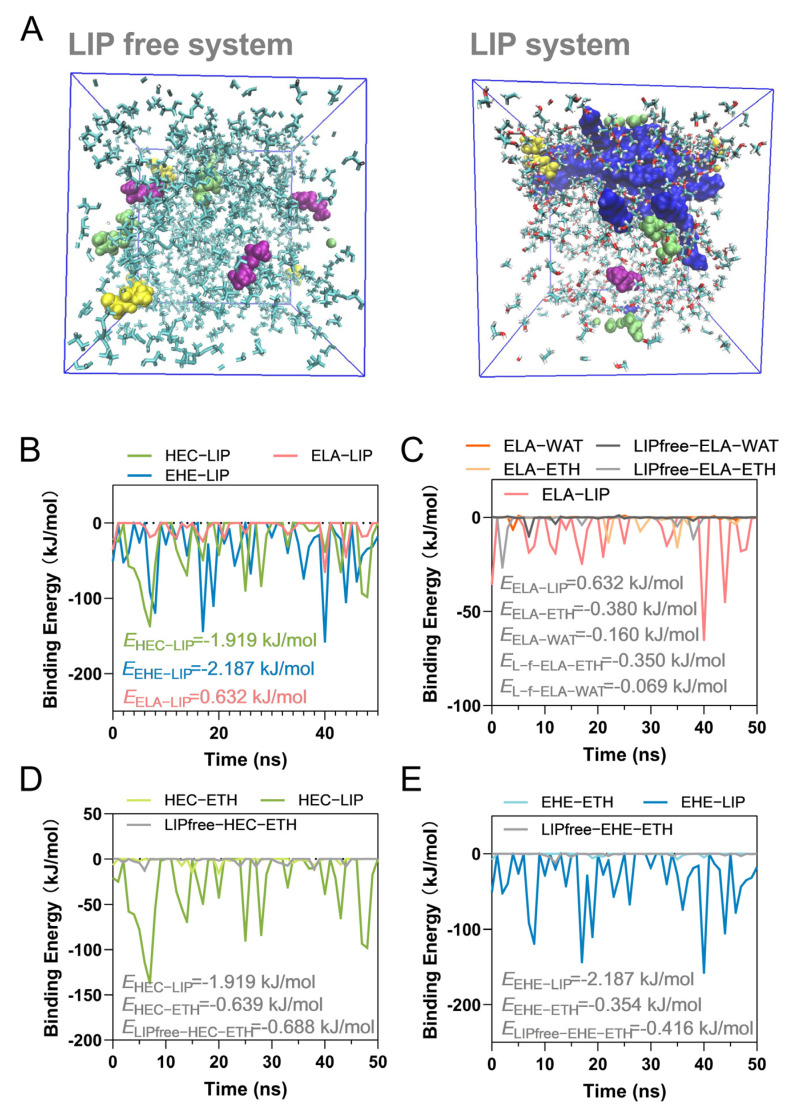
(**A**) Motion trajectory section of the ethanol–lipid–aroma compounds system; (**B**) the binding energy of baijiu aroma compounds and lipid; (**C**) the binding energy of ethyl lactate and solvent molecule (ELA is for ethyl lactate); (**D**) the binding energy of heptanoic acid and solvent molecule (HEC is for heptanoic acid); (**E**) the binding energy of ethyl hexanoate and solvent molecule (EHE is for ethyl hexanoate).

The 53% ABV system and the 53% ABV + peanut lipid system were established, respectively, and an equivalent amount of compound molecules was added. As can be observed from the molecular motion trajectory (Figure 3), in the 53% ABV system, the ethanol molecules showed an aggregating tendency, forming ethanol clusters, with the compound molecules encapsulated within these clusters. This led to an increase in the solubility of the compounds and a consequent reduction in their release intensity. In the 53% ABV–peanut lipid system, the peanut lipid molecules were present in an aggregated state, and both the ethanol and all the baijiu aroma compound molecules aggregated around the peanut lipid molecules, whether they were lipophilic or hydrophilic. In the case of lipophilic compounds, the binding ability of the lipid molecules with the ethanol molecules was greater than that with the compounds. Ethanol and lipid had an antagonistic effect on the release intensity of the aroma compounds. For the hydrophilic compounds, the lipid could interact with the ethanol, weakening the interaction force between the ethanol and the water molecules, releasing more water to combine with the compounds, and thus weakening their release intensities.

The results of the MMPBSA-binding free energy showed that in the 53% ABV solution, the binding energy between the compound and the ethanol was −0.977 kJ/mol, while in the 53% ABV–peanut lipid system, the binding energy between them decreased to −0.742 kJ/mol. The peanut lipid molecules reduced the binding energy between ethanol and the baijiu flavor substances. At the same time, the binding energy between the lipid molecules and the ethanol molecules was −2.18 kJ/mol. This indicates that there is a strong interaction between the peanut lipid molecules and ethanol, resulting in a weakened retention ability of the ethanol molecules for the compound molecules.

The interaction energies of three aroma compounds with matrix molecules were calculated. After adding peanut lipid, the binding energies between ethyl lactate and the water and ethanol decreased (with ethanol: −0.380 to −0.350 kJ/mol, with water: −0.160 to −0.069 kJ/mol). The lipid–ethyl lactate binding energy was 0.632 kJ/mol. There was no spontaneous interaction between them. In 53% ABV–lipid solution, heptanoic acid-lipid interaction energy (−1.919 kJ/mol) was greater than that with ethanol (−0.639 kJ/mol). The addition of the lipid slightly reduced the interaction energy of heptanoic acid -ethanol (−0.688 kJ/mol in the 53% ABV solution). For ethyl hexanoate, its binding energy with the lipid molecule was −2.187 kJ/mol, and the binding energy with the ethanol molecule was −0.354 kJ/mol. Compared with the results in the control group of 53% ABV, the addition of peanut lipid weakened the binding energy of ethyl hexanoate-ethanol (−0.416 kJ/mol in the 53% ABV solution). The release intensity of the compound in the solution is the result of the balance of multiple forces. For ethyl hexanoate, the effects of lipid and ethanol effects were partially offset. The lipid in the exogenous food can interacts with both hydrophobic compounds and ethanol molecules, weakening the forces between the ethanol and water molecules- aroma compound molecules, causing 1 + 1 < 2 effect.

The interaction forces between the compound and three peanut lipid acylglycerols were studied, respectively (Table 3). The interaction enthalpy changes between ethyl hexanoate and OOL and OLL was greater than that with POL. The interaction force between heptanoic acid and the three acylglycerols was relatively small, and its interaction force with OLL and POL was slightly greater than that with OOL. OOL is the component with the highest content in peanut acylglycerol, which makes the binding energy between ethyl heptanoate and peanut lipid smaller than that of ethyl hexanoate-peanut lipid. Van der Waals force (or hydrogen bond) is the main interaction force between the baijiu flavor substances and the peanut lipid molecules.

### 3.3. Influence of the Oral Coating Formed by Peanut Lipid on Baijiu Aftertaste

**Figure 4:**
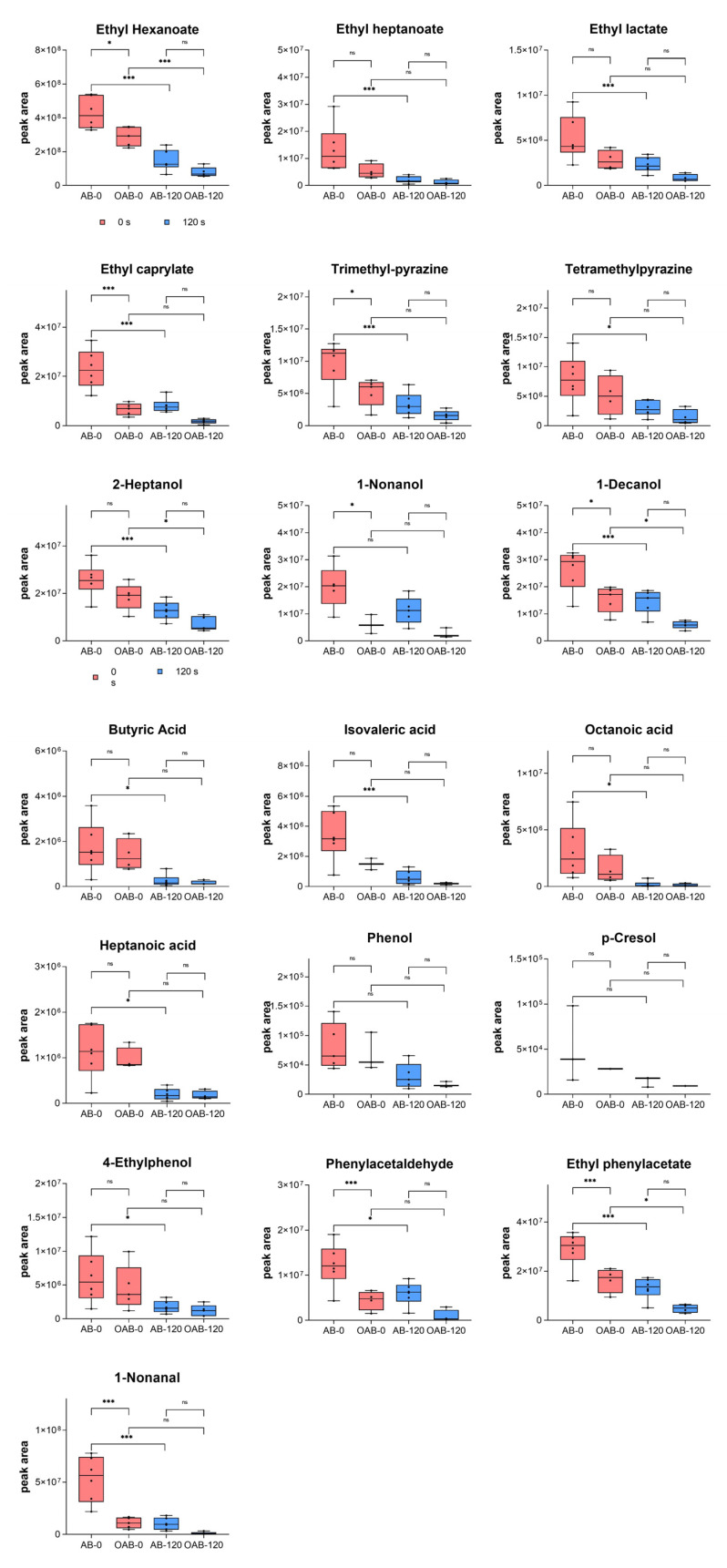
Release intensity of compounds at 0 s and 120 s in the in vivo experiment of baijiu aroma compounds under the influence of mouth coating formed by peanut lipid. The “*” represents *p* < 0.05, “***” represents *p* < 0.001, and “ns” represents no significant difference in the multiple *t*-test.

The mass transfer coefficient is the main factor affecting the aroma release in the oral cavity [22]. The Intraoral SPME method was used to study this process in baijiu drinking (Figure 4). In the lipid-free condition, the release intensity of the compound decreased non-linearly, indicating the retention effect of the oral mucosa to baijiu, which significantly affects its aftertaste [23]. The release pattern of baijiu aroma compounds in the oral cavity is driven by the retention ability of oral mucosa, ranked as follows: alcohols > aromatic compounds > esters = pyrazines > phenols > aldehydes > acids.

Figure 4 presents a comparison between 0 s and 120 s in the lipid-free and lipid-coating groups. At 0 s, the lipid weakened the release intensity of all target compounds, consistent with the in vitro experiments. Notably, the release intensities of ethyl hexanoate, nonanal, 2,3,5-trimethylpyrazine, ethyl octanoate, phenylacetaldehyde, nonanol, decanol, and ethyl phenylacetate decreased significantly. Particularly, nonanal with rose and orange aroma decreased by 79.5%, and phenylacetaldehyde with sweet aroma decreased by 63.5%. Aldehydes have a high retention rate in the lipid, followed by the esters. The release intensities of ethyl heptanoate and ethyl octanoate decreased by 60.6% and 71.2%, respectively, while ethyl hexanoate had a relatively low decrease proportion of 32.3%. The decrease in phenolic compounds was minimal, with phenol decreasing by only 15.3%.

The intensity reductions at 0 s under the influence of peanut lipid in vitro and in vivo were similar, especially for phenol, phenylacetaldehyde, 4-ethylphenol, 1-decanol, ethyl phenylacetate, butyric acid, octanoic acid, heptanoic acid, 2-heptanol, tetramethyl pyrazine, trimethyl pyrazine, p-cresol, and ethyl hexanoate. These could be retained in the mouth coating and contribute to aftertaste perception. For alcohols and esters, their in vivo decrease proportions were slightly lower than in vitro due to oral mucosa retention. However, for some compounds like nonanal, ethyl heptanoate, and ethyl octanoate (with relatively high log *p* values), the in vivo decrease proportions under the influence of lipid were much greater than those in the headspace experiment, possibly due to the spitting process in the experimental procedure. Compared to the in vitro experiment, many other factors influenced aroma release in vivo. After the formation of the mouth coating, spitting out the baijiu washes away part of the lipid and flavor substances, similarly to swallowing. To ensure sufficient compound–lipid contact in the oral cavity, the rinsing time of baijiu was 15 s, much longer than the actual consumption process. This would lead to a significant loss of highly hydrophobic compounds; however, their physicochemical properties allow them to persist in the mouth coating.

**Figure 5:**
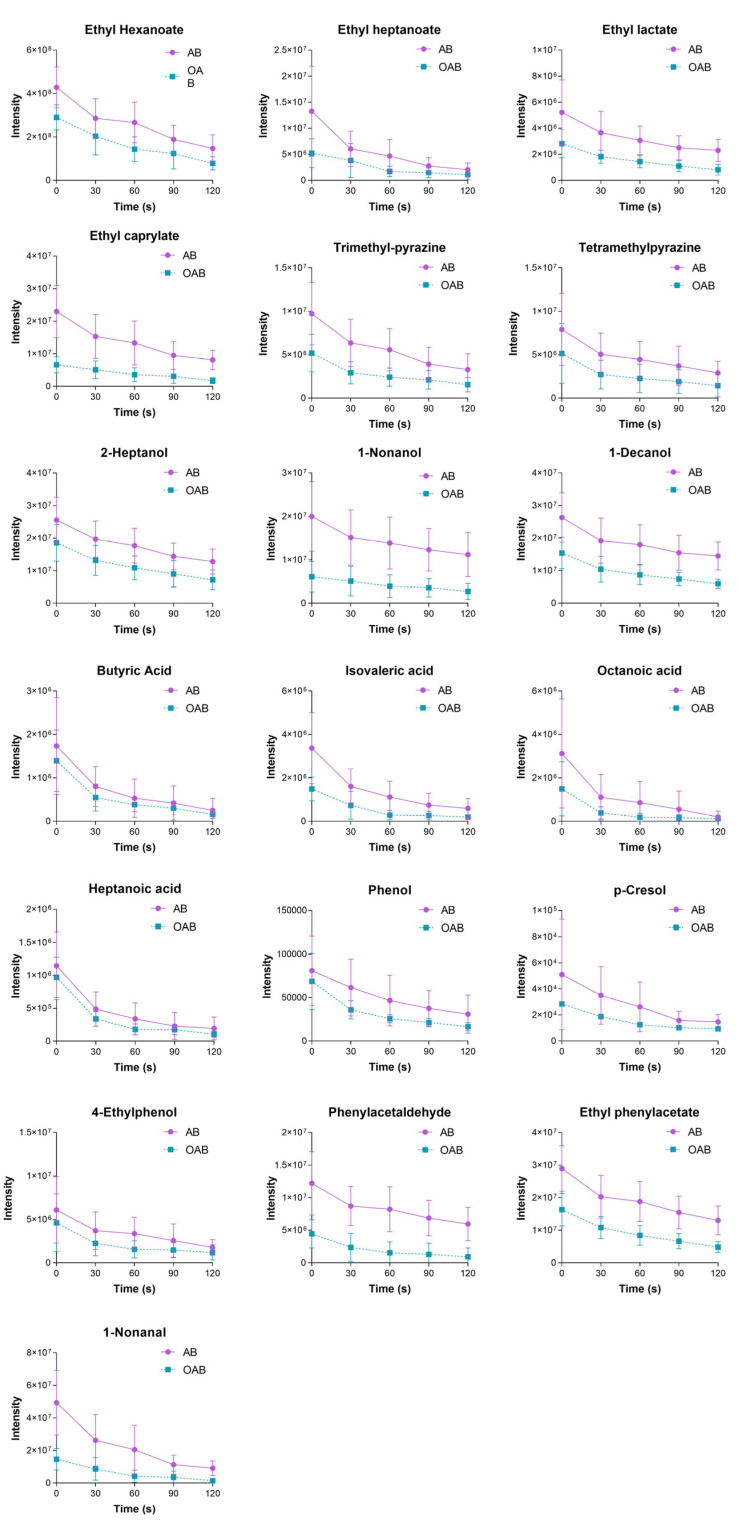
Intensity changes in baijiu aroma compound in the vivo experiment under the influence of mouth coating formed by peanut lipid.

The dynamic changes in the compounds at equidistant time nodes differed from the control group and did not change linearly, indicating that the release of baijiu flavor substances in the oral cavity was strongly and unevenly affected by the mouth coating forming by peanut lipid [20]. The influence of lipid on baijiu’s aftertaste was mainly in the initial drinking stage. Without lipid, crucial aroma compounds of baijiu were rapidly consumed within the first 30 s, faster than that in the lipid-containing system. Compared with the lipid-free system, the reductions in ethyl heptanoate, ethyl lactate, nonanal, 2,3,5-trimethylpyrazine, ethyl octanoate, tetramethyl pyrazine, butyric acid, phenylacetaldehyde, nonanol, isovaleric acid, phenol, octanoic acid, p-cresol, and 4-ethylphenol in the lipid-coating system were no longer significant. Ethyl heptanoate, ethyl lactate, and ethyl octanoate are the key esters in baijiu [1]. In the experiment conducted on wine, an enzymatic hydrolysis reaction was believed to break down esters into corresponding acids and alcohols [23]. However, in baijiu, the high concentration of ethanol inactivates the enzyme, preventing the esterase reaction [16]. In this experiment, the decrease in esters were not significant. In both the lipid-containing system and the oil-free system, the compound intensities at 0 s had significant differences (*t*-test, *p* < 0.05), but at 120 s, the release intensities of all compounds were no longer significant (t-test, multiple *t*-test, *p* > 0.05, Figure 5). This indicates that the lipid can reduce the release speed of the important compounds in baijiu and prolong the aftertaste’s duration. The oral coating formed by peanut lipid can retain the compounds and slowly release them during the subsequent aftertaste.

**Figure 6:**
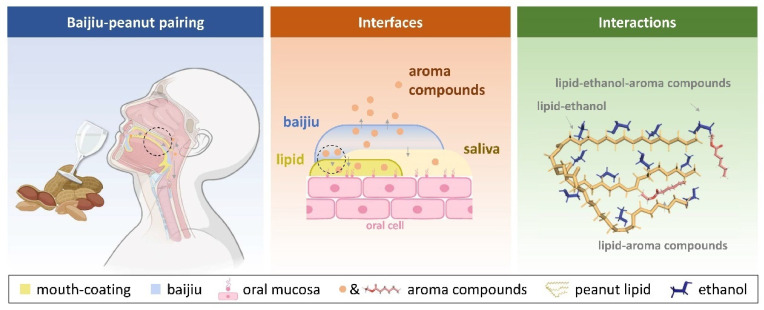
Schematic diagram of the influence of peanut lipid on the release of flavor substances in baijiu. The circles in the figure indicate the key points of focus in this study.

Moreover, the ethanol in baijiu could also be retained by mouth coating. Due to the Marangoni effect of ethanol, the presence of ethanol can sustain the continuous release of flavor substances in baijiu at a stable flow rate until they are consumed [14,15]. Given that ethanol exhibits surface activity, in the water (saliva)–ethanol solution, it preferentially resides at the air/liquid interface, thus diminishing the surface tension. During the dynamic process, the headspace becomes diluted, and the ethanol at the interface commences to evaporate. As the surface ethanol concentration declines, some depletion regions will emerge at the liquid surface with higher surface tension, promoting the molecules in the adjacent low surface tension regions to migrate towards the high surface tension regions. Driven by the surface tension gradient, the ethanol molecules in the lower layer ascend to supplement the liquid surface. Simultaneously, they carry the aroma molecules to escape into the gas phase at a constant rate. In the relevant dynamic equilibrium experiment, when the alcohol content of the system exceeds 6% ABV, the headspace concentration of the ethanol solution is higher than that of the aqueous solution with the same compound concentration. Moreover, its headspace concentration remains essentially stable as the ethanol concentration declines [24]. Consequently, even during the aftertaste stage, the continuous rinsing of the oral cavity by saliva, which dilutes the ethanol concentration, can still maintain the aftertaste intensity and duration of the baijiu aroma compounds to a certain degree. The lipid in the peanut can extend the duration of baijiu aftertaste in two manners (Figure 6).

## 4. Conclusions

Peanut lipid can significantly decrease the release intensity of flavor substances. In the baijiu–peanut pairing, under influence of ethanol, the inhibitory effect of peanut lipid on the baijiu aftertaste intensity is weakened because of the antagonistic effect of ethanol and lipid on the solubility of compounds, and it was highly related with the log *p*, boiling point, and vapor pressure of the aroma compounds. Through the detection of equilibrium constant, the inhibitory effect was found to be concentration-dependent. Molecular dynamics simulation revealed a strong interaction between peanut lipid and ethanol (Ebinding = −2.181 kJ/mol), resulting in a 1 + 1 < 2 effect. For the in vivo condition, the Intraoral SPME revealed that the retention order of important flavor substances by mouth coating formed by peanut lipid is as follows: aldehydes > esters > acids ≈ benzene ring compounds ≈ alcohols > nitrogen-containing compounds > phenols. They continuously play a crucial role in the perception of aftertaste. The interaction between peanut lipid and both the baijiu aroma compounds and ethanol, in conjunction with the Marangoni effect between ethanol and baijiu flavor substances, collaboratively reinforces the prolonged aftertaste of baijiu. This research reveals the mechanism through which peanut lipid extends the aftertaste duration of baijiu in the baijiu–peanut pairing. By means of carefully choosing and adjusting the accompanying food, it might be feasible to adjust the baijiu aftertaste and improve consumers’ sensory perception. It furnishes a scientific foundation for comprehending the intricate flavor experience during the actual drinking process.

## Figures and Tables

**Table 1:** Target compound information.

	CAS	Aroma	Log *p*	Boiling Point	Vapor Pressure	Water Solubility	Henery’s Law Constant	Concentration of the In Vitro Experiment (mg/L)	Concentration of Added Aromatic Substances in Baijiu (mg/L)
Ethyl hexanoate	123-66-0	sweet, fruity	2.83	167	1.80	309	7.23 × 10^−4^	50	-
2-Heptanol	543-49-7	lemon, floral	2.29	161	1.23	3.57 × 10^3^	2.34 × 10^−5^	100	50
Ethyl heptanoate	106-30-9	pineapple, rum	3.37	187	0.68	102	9.60 × 10^−4^	50	-
Ethyl lactate	97-64-3	fruity, buttery	−0.19	154	3.75	4.73 × 10^−5^	4.82 × 10^−5^	300	200
1-Nonanal	124-19-6	floral, lemon	3.27	191	0.37	132	4.93 × 10^−4^	100	50
Ethyl caprylate	106-32-1	fruity, wine	3.81	209	0.24	33.4	1.27 × 10^−3^	50	-
Trimethyl pyrazine	14667-55-1	potato, roast	0.95	172	1.45	1.52 × 10^−4^	3.92 × 10^−6^	10	-
Tetramethyl pyrazine	1124-11-4	coffee, roast	1.56	190	0.15	7.01 × 10^3^	4.33 × 10^−6^	10	-
Butyric acid	107-92-6	cheese, butter	1.07	164	1.65	6.61 × 10^−4^	5.35 × 10^−7^	700	100
Phenylacetaldehyde	122-78-1	green, floral	1.78	195	0.35	3.03 × 10^3^	5.48 × 10^−6^	500	50
1-Nonanol	124-19-6	floral, lemon	3.27	191	0.37	132	4.93 × 10^−4^	100	20
Isovaleric acid	503-74-2	cheese, pungent	1.16	177	0.44	2.92 × 10^−4^	1.28 × 10^−6^	700	100
1-Decanol	112-30-1	fatty, floral	4.57	231	0.01	28.2	5.47 × 10^−5^	200	50
Ethyl phenylacetate	101-97-3	honey, rose	2.50	227	0.09	729	1.88 × 10^−5^	100	-
Phenol	108-95-2	plastic, rubber	1.48	182	0.35	2.62 × 10^−4^	5.61 × 10^−7^	100	-
Heptanoic acid	111-14-8	sour, sweat	2.37	222	0.12	1.96 × 10^3^	2.26 × 10^−6^	400	50
Octanoic acid	124-07-2	fatty, waxy	3.03	239	0.05	496	3.00 × 10^−6^	400	50
p-Cresol	106-44-5	pungent, stable	1.94	202	0.11	9.25 × 10^3^	6.19 × 10^−7^	100	-
4-Ethylphenol	123-07-9	leather, spice	2.47	218	0.04	2.35 × 10^3^	8.21 × 10^−7^	100	20

**Table 2:** Changes in equilibrium constant under the influence of lipid.

	Water Solution	53% ABV	53% ABV + 4% Lipid	53% ABV + 8% Lipid
Ethyl caproate	0.1494 ± 0.0345 ^c^	0.0574 ± 0.0025 ^a^	0.0267 ± 0.0035 ^b^	0.0179 ± 0.0013 ^b^
2-Heptanol	0.0405 ± 0.0034 ^a^	0.0375 ± 0.0035 ^a^	0.0283 ± 0.0034 ^ab^	0.0212 ± 0.0020 ^b^
Ethyl heptanoate	0.1669 ± 0.0438 ^a^	0.1356 ± 0.0146 ^b^	0.0252 ± 0.0030 ^c^	0.0193 ± 0.0014 ^c^
Ethyl lactate	0.0050 ± 0.0004 ^c^	0.1004 ± 0.0089 ^a^	0.0362 ± 0.0047 ^b^	0.0100 ± 0.0005 ^c^
1-Nonanal	0.1818 ± 0.0038 ^a^	0.1143 ± 0.0110 ^b^	0.0581 ± 0.0065 ^c^	0.0181 ± 0.0018 ^d^
Ethyl caprylate	0.1193 ± 0.0145 ^a^	0.1064 ± 0.0083 ^a^	0.0429 ± 0.0048 ^b^	0.0151 ± 0.0013 ^c^
Trimethyl pyrazine	0.0087 ± 0.0010 ^c^	0.0651 ± 0.0038 ^a^	0.0482 ± 0.0052 ^b^	0.0327 ± 0.0030 ^b^
Tetramethyl pyrazine	0.0092 ± 0.0010 ^c^	0.0738 ± 0.0043 ^a^	0.0423 ± 0.0043 ^b^	0.0456 ± 0.0024 ^b^
Butyric acid	0.0180 ± 0.0021 ^c^	0.1029 ± 0.0082 ^a^	0.0705 ± 0.0056 ^b^	0.0227 ± 0.0038 ^c^
Phenylacetaldehyde	0.0015 ± 0.0002 ^c^	0.2681 ± 0.0261 ^a^	0.0513 ± 0.0050 ^b^	0.0113 ± 0.0011 ^c^
1-Nonanol	0.0150 ± 0.0003 ^c^	0.1166 ± 0.0179 ^a^	0.0742 ± 0.0087 ^b^	0.0179 ± 0.0023 ^c^
Isovaleric acid	0.0152 ± 0.0002 ^b^	0.0440 ± 0.0064 ^a^	0.0358 ± 0.0034 ^a^	0.0090 ± 0.0007 ^b^
1-Decanol	0.0350 ± 0.0003 ^a^	0.0608 ± 0.0071 ^a^	0.0357 ± 0.0029 ^b^	0.0182 ± 0.0022 ^c^
Ethyl phenylacetate	0.0014 ± 0.0001 ^c^	0.0560 ± 0.0078 ^a^	0.0369 ± 0.0038 ^b^	0.0450 ± 0.0024 ^ab^
Phenol	0.0300 ± 0.0035 ^a^	0.0220 ± 0.0018 ^ab^	0.0168 ± 0.0020 ^ab^	0.0094 ± 0.0009 ^b^
Heptanoic acid	0.0136 ± 0.0005 ^b^	0.0540 ± 0.0052 ^a^	0.0268 ± 0.0028 ^a^	0.0095 ± 0.0003 ^b^
Octanoic acid	0.0125 ± 0.0015 ^c^	0.0638 ± 0.0058 ^a^	0.0355 ± 0.0017 ^b^	0.0138 ± 0.0005 ^c^
p-Cresol	0.0004 ± 0.0001 ^c^	0.0499 ± 0.0038 ^a^	0.0299 ± 0.0032 ^b^	0.0102 ± 0.0006 ^c^
4-Ethylphenol	0.0333 ± 0.0028 ^a^	0.0208 ± 0.0020 ^ab^	0.0158 ± 0.0012 ^b^	0.0138 ± 0.0009 ^b^

The “^a^” “^b^” “^c^” “^d^” after the numbers represents the significant level of the equilibrium constant for the same compound in different solutions.

**Table 3:** Interaction energy between molecules.

	*E*_solv, polar_	*E*_solv, nonpolar_	*E*_MM_	*E*_ele_	*E*_vdw_
ELA-OOL	2.322	−1.437	−5.020	−0.298	−4.722
ELA-OLL	2.651	−1.148	−5.693	−0.485	−5.208
ELA-POL	1.154	−1.053	−2.793	−0.064	−2.729
EHE-OOL	4.750	−5.604	−21.408	−0.386	−19.001
EHE-OLL	4.595	−5.103	−19.605	−0.408	−19.197
EHE-POL	2.775	−3.340	−11.735	−0.199	−11.536
HEC-OOL	3.679	−4.554	−14.615	−0.122	−14.493
HEC-OLL	3.629	−4.661	−15.458	−0.326	−15.132
HEC-POL	4.901	−4.701	−16.679	−0.372	−16.307

EHE is for ethyl hexanoate. ELA is for ethyl lactate. HEC is for heptanoic acid. OOL, OLL, and POL is the triglyceride of peanut lipid, O is for oleic acid, L is for linoleic acid, and P is for palmitic acid.

## Data Availability

The datasets presented in this article are not readily available because the data are part of an ongoing study.

## References

[1] Liu H., Sun B. (2018). Effect of Fermentation Processing on the Flavor of Baijiu. J. Agric. Food Chem..

[2] Chen L., Zhao Y., Chen X., Zhang Y., Li H., Zhao D., Wang B., Ye X., Sun B., Sun J. (2024). Peanut Pairing Baijiu: To Enhance Retronasal Aroma Intensity while Reducing Baijiu Aftertaste. J. Agric. Food Chem..

[3] Chen L., Yang Y., Hu X., Li H., Zhao D., Wang B., Ye X., Zhang Y., Sun B., Sun J. (2025). Unraveling the role of peanut protein in baijiu-peanut pairing flavor complexity: A focus on ethanol-induced denaturation. Food Chem..

[4] Carrín M.E., Carelli A.A. (2010). Peanut oil: Compositional data. Eur. J. Lipid Sci. Technol..

[5] Tarrega A., Yven C., Sémon E., Salles C. (2011). In-mouth aroma compound release during cheese consumption: Relationship with food bolus formation. Int. Dairy J..

[6] Guichard E. (2002). Interactions between flavor compounds and food ingredients and their influence on flavor perception. Food Rev. Int..

[7] de Roos K.B., Shahidi F., Weenen H. (2006). How lipids influence flavor perception. Food Lipids: Chemistry, Flavor, and Texture.

[8] Guichard E., Galindo-Cuspinera V., Feron G. (2018). Physiological mechanisms explaining human differences in fat perception and liking in food spreads-a review. Trends Food Sci. Technol..

[9] van Ruth S.M., King C., Giannouli P. (2002). Influence of lipid fraction, emulsifier fraction, and mean particle diameter of oil-in-water emulsions on the release of 20 aroma compounds. J. Agric. Food Chem..

[10] Seuvre A.M., Voilley A. (2017). Physico-Chemical Interactions in the Flavor-Release Process.

[11] Mao L., Roos Y.H., Biliaderis C.G., Miao S. (2017). Food emulsions as delivery systems for flavor compounds: A review. Crit. Rev. Food Sci. Nutr..

[12] Camacho S., van Riel V., de Graaf C., van de Velde F., Stieger M. (2014). Physical and sensory characterizations of oral coatings of oil/water emulsions. J. Agric. Food Chem..

[13] Boisard L., Andriot I., Arnould C., Achilleos C., Salles C., Guichard E. (2013). Structure and composition of model cheeses influence sodium NMR mobility, kinetics of sodium release and sodium partition coefficients. Food Chem..

[14] Tsachaki M., Linforth R.S.T., Taylor A.J. (2005). Dynamic Headspace Analysis of the Release of Volatile Organic Compounds from Ethanolic Systems by Direct APCI-MS. J. Agric. Food Chem..

[15] Taylor A.J., Tsachaki M., Lopez R., Morris C., Ferreira V., Wolf B. (2010). Odorant Release from Alcoholic Beverages. Flavors in Noncarbonated Beverages.

[16] Chen L., Wang Z., Liao P., Li A., Zhang Y., Li H., Sun J. (2021). The effect of saliva on the aroma release of esters in simulated baijiu under the impact of high ethanol concentration. J. Food Compos. Anal..

[17] Lesme H., Alleaume C., Bouhallab S., Famelart M.H., Marzin C., Lopez-Torres L., Prost C., Rannou C. (2020). Aroma-retention capacities of functional whey protein aggregates: Study of a strawberry aroma in solutions and in fat-free yogurts. Food Res. Int..

[18] Savary G., Hucher N., Petibon O., Grisel M. (2014). Study of interactions between aroma compounds and acacia gum using headspace measurements. Food Hydrocoll..

[19] Yu Y., Chen S., Nie Y., Xu Y. (2022). Optimization of an intra-oral solid-phase microextraction (SPME) combined with comprehensive two-dimensional gas chromatography-time-of-flight mass spectrometry (GC x GC-TOFMS) method for oral aroma compounds monitoring of Baijiu. Food Chem..

[20] Yu Y., Nie Y., Chen S., Xu Y. (2023). Characterization of the dynamic retronasal aroma perception and oral aroma release of Baijiu by progressive profiling and an intra-oral SPME combined with GC × GC-TOFMS method. Food Chem..

[21] Chen L., Yan R., Zhao Y., Sun J., Zhang Y., Li H., Zhao D., Wang B., Ye X., Sun B. (2023). Characterization of the aroma release from retronasal cavity and flavor perception during baijiu consumption by Vocus-PTR-MS, GC×GC-MS, and TCATA analysis. LWT.

[22] Linforth R., Martin F., Carey M., Jim D., Taylor A. (2003). Retronasal Transport of Aroma Compounds. J. Agric. Food Chem..

[23] Lyu J., Fu J., Chen S., Xu Y., Nie Y., Tang K. (2022). Impact of tannins on intraoral aroma release and retronasal perception, including detection thresholds and temporal perception by taste, in model wines. Food Chem..

[24] Munoz-Gonzalez C., Perez-Jimenez M., Criado C., Pozo-Bayon M.A. (2019). Effects of Ethanol Concentration on Oral Aroma Release After Wine Consumption. Molecules.

